# Low 25(OH)Vitamin D levels are associated with nutritional risk and disease severity in outpatient individuals with advanced chronic liver disease

**DOI:** 10.1017/jns.2026.10113

**Published:** 2026-06-16

**Authors:** Josile Maria da Conceição Albuquerque, José Israel Rodrigues Junior, Aryana Isabelle de Almeida Neves Siqueira, Juliana Célia de Farias Santos, João Araújo de Barros-Neto, Nassib Bezerra Bueno, Fabiana Andréa Moura

**Affiliations:** 1 Faculdade de Nutrição, Federal University of Alagoas, Brazil; 2 Pós-Graduação em Nutrição (PPGNUT), Federal University of Alagoas, Brazil; 3 Programa de Pós-Graduação em Ciências Médicas, Federal University of Alagoas, Brazil

**Keywords:** Ascites, Avitaminosis, End stage liver disease, Liver cirrhosis, Nutritional status

## Abstract

The aim of this cross-sectional study was to evaluate the association between serum 25-hydroxyvitamin D [25(OH)VitD], disease severity, nutritional and functional status in individuals with advanced chronic liver disease (ACLD). Clinical assessment included Child-Pugh, Model for End-Stage Liver Disease-Sodium (MELD-Na), and MELD 3.0 scores, as well as the presence of ascites. Nutritional status was assessed using disease-specific screening tools, anthropometry, body composition measures, including Dual-Energy X-ray Absorptiometry (DXA), sarcopenia and Liver Frailty Index (LFI). A total of 47 outpatients with ACLD were evaluated. 25(OH)VitD insufficiency (<30 ng/mL) prevalence was 76.6%. After multivariate adjustment, insufficiency was significantly associated with Child-Pugh B/C (OR = 8.247; *p* = 0.013), ascites (OR = 15.382; *p* = 0.016), and nutritional risk by RFH-NPT (OR = 13.168; *p* = 0.015). No independent associations were found with sarcopenia or frailty. Notably, ascites emerged as the pivotal link between clinical decompensation and nutritional vulnerability; its presence is a primary RFH-NPT criterion, which remained the only nutritional marker independently associated with vitamin D depletion. In conclusion, 25(OH)VitD insufficiency was associated with advanced disease severity and nutritional vulnerability. Patients at nutritional risk had 13.2 times higher odds of 25(OH)VitD insufficiency, while those with ascites and Child-Pugh B/C showed 15.4 and 8.2 times higher odds, respectively. These findings emphasise that 25(OH)VitD levels are more closely linked to early nutritional risk than to established sarcopenia and frailty in this population.

## Graphical abstract

**Figure:**
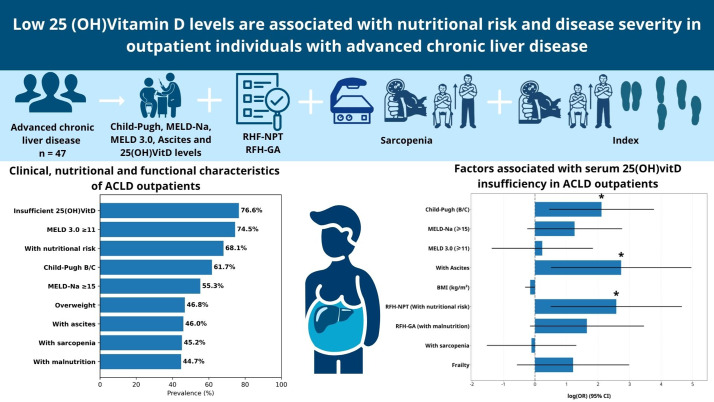

## Introduction

Active vitamin D [1,25(OH)_2_VitD] serves a variety of functions in the body including modulating of the immune system, cell differentiation and proliferation, and inflammatory processes.^(1)^ To become biologically active, VitD undergoes two sequential hydroxylation reactions: the first occurs in the liver, forming 25(OH)VitD (most abundant in circulation),^(2)^ and the second takes place in the kidneys, where it is converted into the active form, 1,25(OH)_2_VitD.^(3)^

Since its metabolism occurs in the liver, low 25(OH)VitD levels are commonly observed in patients with advanced chronic liver disease (ACLD).^(4,5)^ This is particularly concerning given that 25(OH)VitD has a supposed suppressive effect on collagen production in hepatic stellate cells, which may regulate the fibrotic process.^(3,6)^

The close association between low 25(OH)VitD levels, malnutrition, and sarcopenia is widely recognised in children, adults, and the elderly, as these conditions can develop from various factors and occur successively or simultaneously. Impaired macronutrient and micronutrient intake and absorption, combined with increased gluconeogenesis from amino acids, favours muscle depletion and elevates the risk of sarcopenia.^(7,8)^ Although the mechanisms by which 25(OH)VitD acts on muscle are not fully clear, there is evidence that its receptor, the vitamin D receptor (VDR), induces cell differentiation and proliferation. It does this by positively regulating growth factors such as Insulin-like Growth Factor-1 (IGF-1) and follistatin, while also inhibiting the expression of myostatin, a key regulator of muscle growth.^(5)^

The strong connection between low 25(OH)VitD levels and sarcopenia has been consistently investigated in recent years. For instance, patients with decompensated cirrhosis (Child-Pugh B and C) and sarcopenia, or very low levels of this vitamin (<10 ng/mL), had a worse clinical prognosis.^(9)^ Other studies also observed that 25(OH)VitD levels <25 ng/mL were independently associated with sarcopenia in individuals with ACLD.^(10)^ However, despite these results, there is a lack of uniformity among authors regarding the diagnosis of sarcopenia in individuals with ACLD. In some cases, the method for identifying muscle mass is not described, or the authors use techniques that are not appropriate for this population, such as bioimpedance. To address this methodological inconsistency and ensure diagnostic rigour, our study utilised Dual-Energy X-ray Absorptiometry (DXA), a recommended method for assessing skeletal muscle mass in this patient population.

Therefore, this study aimed to evaluate the association between serum 25(OH)VitD levels and disease severity in individuals with ACLD. Furthermore, we investigated the relationship between 25(OH)VitD and nutritional and functional status, specifically focusing on nutritional risk, sarcopenia, and physical frailty.

## Materials and methods

### Study design and setting

This is a cross-sectional study derived from a larger research project conducted at the Infectious and Parasitic Diseases Department of Professor Alberto Antunes University Hospital, Maceió, Alagoas, Brazil. Data were collected from October 2022 to November 2023. The study design and reporting followed the guidelines of the Strengthening the Reporting of Observational Studies in Epidemiology (STROBE).

### Sample and study groups

The study included patients aged 18 to 70 years, of both sexes, diagnosed with ACLD and portal hypertension. Portal hypertension was defined by the presence of ascites, splenomegaly, esophagogastric varices, or portosystemic collaterals (patent paraumbilical vein, splenorenal collaterals, dilated left gastric veins, and short veins).

Exclusion criteria were (a) neoplasia; (b) acute liver failure; (c) pregnant and lactating women; (d) human immunodeficiency virus infection; (e) patients listed for liver transplantation due to special conditions (intractable pruritus, recurrent cholangitis, refractory ascites, persistent hepatic encephalopathy); and (f) history of organ failure affecting nutritional status, such as renal replacement therapy, respiratory, and cardiac failure.

### Sample size calculation

This was an exploratory study derived from a project that originally aimed to identify the prevalence of sarcopenia among liver transplant (LT) candidates (MELD-Na ≥ 15). A relative risk of 3 for sarcopenia prevalence was expected, with a baseline prevalence of 25% in the control group (patients with ACLD but without LT indication). Assuming 80% power and a 5% alpha level, 19 patients were required in each group (MELD-Na ≤ 14 and MELD-Na ≥ 15). The final sample of 47 patients exceeded the minimum requirement, ensuring adequate power for the primary objectives of the parent study.

### Data collection and assessments

#### Liver disease severity assessment

The severity of liver disease was assessed using the MELD-Na (≤14 and ≥15), MELD 3.0 (≤10 and ≥11), and Child-Pugh scores (A and B/C). All scores were determined by a specialised medical professional based on clinical evaluation and laboratory test results obtained at the time of consultation.

#### Sociodemographic and clinical data

Sociodemographic and clinical data, including personal and current disease history, presence of signs and symptoms, lifestyle habits, disease aetiology, and time of diagnosis, were collected using a standardised form.

#### Nutritional and functional assessment

To ensure a rigorous nutritional and functional assessment appropriate for this patient population, we used tools and techniques specifically validated for individuals with advanced chronic liver disease.

Nutritional risk was assessed using the Royal Free Hospital Nutritional Prioritising Tool (RFH-NPT). Patients were classified as having nutritional risk if their RFH-NPT score was ≥1 point. Nutritional status was identified using the Royal Free Hospital Global Assessment (RFH-GA) and classified as malnourished based on the tool’s flowchart. Arm circumference (AC) and triceps skinfold (TSF) were measured and used to calculate RFH-GA.

Body Mass Index (BMI) was calculated using the patients’ current weight. For those with ascites or oedema, an estimated weight was obtained by subtracting a percentage of weight based on the severity of ascites (5% for mild, 10% for moderate, and 15% for severe)^(8)^ or by reducing 1, 3, or 6 kg for mild, moderate, or severe lower limb oedema, respectively.^(11)^ The nutritional status categories were underweight, adequate weight, overweight, and obesity, based on specific cutoffs for adults and older adults.^(12,13)^ BMI was also used for the RFH-GA tool. Skeletal muscle mass was evaluated by DXA, performed with the patient in a supine position with legs extended, feet together, and arms at their side. Upper and lower limb muscle mass were summed to determine Appendicular Skeletal Muscle Mass (ASMM), and its index (ASMMI) was calculated as ASMM/height^2^. Muscle mass reduction was defined as ASMM < 20 kg or ASMMI < 7 kg/m^2^ for men, and ASMM < 15 kg or ASMMI < 5.5 kg/m^2^ for women.^(14)^

Muscle strength and functional capacity were assessed using handgrip strength (HGS) and the five-times sit-to-stand test. HGS was measured three times on the dominant hand, with the average used to classify decreased muscle strength (<27 kgf for men and <16 kgf for women), according to Brazilian studies.^(15,16)^ Low muscle strength in the sit-to-stand test was defined as a time exceeding 15 s for five repetitions.^(14)^ Sarcopenia was diagnosed when both low muscle strength (HGS or sit-to-stand test) and reduced muscle mass (ASMM or ASMMI) were present.^(17)^

Physical frailty was assessed using the Liver Frailty Index (LFI), calculated from handgrip strength, the five-times sit-to-stand test, and a standardised balance test. Scores were determined using the official University of California, San Francisco (UCSF) calculator (https://liverfrailtyindex.ucsf.edu/). For statistical purposes, patients were categorised as ‘Robust’ (LFI <3.2) or ‘Frail’ (≥3.2, including pre-frail and frail individuals).

#### 25(OH)VitD level assessment

Serum 25(OH)VitD levels were assessed on the day of consultation using a chemiluminescence method on fasting samples. Vitamin insufficiency was defined as serum 25(OH)VitD levels <30 ng/mL.

#### Equipment and techniques

Body weight and height were measured using a Filizola® digital scale and a metal anthropometer. AC was measured with a non-extensible tape, while TSF was assessed with a Lange® calliper. ASMM was obtained via DXA analysis using a Lunar Prodigy Primo system (GE HealthCare). HGS was measured using a Jamar® dynamometer. All measurements were performed by trained professionals.

### Efforts to address potential sources of bias

#### Selection bias

To minimise selection bias, clear eligibility criteria were applied to ensure only patients meeting predefined conditions were included. The sample was drawn from a single university hospital, and all eligible adult patients during the study period were invited to participate, avoiding selective inclusion. Efforts were also made to ensure a representative sample, including patients of both sexes and with diverse aetiologies of liver disease.

#### Measurement bias

To reduce measurement bias, standardised protocols were used for all anthropometric and functional assessments. Body composition was measured using DXA, HGS, and the sit-to-stand test. Nutritional parameters were evaluated by trained professionals using calibrated equipment. The same tools and methods were consistently applied to all patients.

#### Confounding factors

Potential confounding factors, such as age, sex, and aetiology of liver cirrhosis, were controlled for in the multivariate regression analysis.

### Ethical considerations

All patients provided written informed consent. The study was conducted in accordance with the ethical guidelines of the 1975 Helsinki Declaration. The protocol was approved by the Ethics Committee on May 26, 2022 (Opinion Number 5432777).

### Statistical analysis

All analyses were conducted using SPSS® version 26.0 (Chicago, IL, USA). Results were expressed as absolute and relative frequencies (*n*/%) or as means and standard deviations. Chi-square or Fisher’s exact tests were applied as appropriate. Logistic regression analyses were performed with 25(OH)VitD insufficiency as the dependent variable. Two adjusted models were constructed: Model 1 included clinical and nutritional variables (Child-Pugh, MELD-Na, Ascites, RFH-NPT, RFH-GA, and frailty) adjusted for sex, age, and aetiology. Model 2 included MELD 3.0 and sarcopenia, adjusted only for age and aetiology, as sex is already incorporated into the MELD 3.0 formula. A significance level of 5% was adopted.

## Results

A total of 47 individuals were evaluated, of whom 76.6% (*n* = 36) had insufficient 25(OH)VitD levels. Two patients were receiving vitamin D supplementation; however, both showed reduced 25(OH)D levels and were therefore included in the analysis.

Of the participants included in the study, 72.3% were male, with a mean age of 48 ± 14.2 years. No statistically significant differences were observed between individuals with adequate or insufficient 25(OH)VitD levels regarding sociodemographic characteristics. Regarding disease aetiology, alcoholic origin was the most prevalent (48.9%), followed by cryptogenic (14.9%), autoimmune (12.8%), and Metabolic Dysfunction–Associated Steatotic Liver Disease (MASLD, 6.4%).

Regarding clinical status, the prevalence of 25(OH)VitD insufficiency was significantly higher among individuals with more severe disease. Specifically, 72.2% of patients with Child-Pugh B or C scores had vitamin insufficiency, compared to 27.8% in the Child-Pugh A group (*p* = 0.012). Additionally, the presence of ascites was significantly associated with insufficiency (58.3% vs. 41.7%; *p* = 0.010), reinforcing the link between low vitamin D levels and clinical decompensation (Table 1).

**Table 1.** Sociodemographic characteristics of patients with advanced chronic liver disease, according to the presence of insufficient 25(OH)VitD levels Table 1 long description.

Variable		25(OH)VitD levels		*p*-Value
	Total	Insufficiency (<30 ng/mL)	Adequate (≥30 ng/mL)	
	*n* (%)	*n* (%)	*n* (%)	
Self-declared race				0.226
White	12 (25.5)	7 (19.4)	5 (45.5)	
Non-White	35 (74.5)	29 (80.6)	6 (54.5)	
Education				0.081
>Elementary school	26 (55.3)	17 (47.2)	9 (81.8)	
<Elementary school or less	21 (44.7)	19 (52.8)	2 (18.2)	
Smoking				0.244
No	35 (74.5)	25 (69.4)	10 (90.9)	
Yes/Former smoker	12 (25.5)	11 (30.6)	1 (9.1)	
Comorbidities				0.999
No	28 (59.6)	21 (58.3)	7 (63.9)	
Yes	19 (40.4)	15 (41.7)	5 (35.7)	
Alcoholic aetiology				0.494
No	24 (51.1)	17 (47.2)	7 (63.6)	
Yes	23 (48.9)	19 (52.8)	4 (36.4)	
Diagnosis time				0.070
<2 years	31 (66.0)	21 (58.3)	10 (90.9)	
≥2 years	16 (34.0)	15 (41.7)	1 (9.1)	
Child-Pugh				0.012
A	18 (38.3)	10 (27.8)	8 (72.7)	
B/C	29 (61.7)	26 (72.2)	3 (27.3)	
MELD-Na				0.181
≤14	21 (44.7)	14 (38.9)	7 (63.6)	
≥15	26 (55.3)	22 (61.1)	4 (3.4)	
MELD 3.0				0.999
≤10	12 (25.5)	3 (27.3)	9 (25)	
≥11	35 (74.5)	8 (72.7)	27 (75)	
Ascites				0.010
No ascites	27 (54.0)	15 (41.7)	12 (85.7)	
With ascites	23 (46.0)	21 (58.3)	2 (14.3)	

MELD-Na: Model for End-Stage Liver Disease-sodium; MELD 3.0: Model for End-Stage Liver Disease 3.0 associated with sex, sodium, and albumin.

Furthermore, from a functional and nutritional standpoint, the prevalence of 25(OH)VitD insufficiency was significantly higher in patients identified at nutritional risk by the RFH-NPT (77.8% vs. 22.2%; *p* = 0.023) (Table 2). No statistically significant associations were found between vitamin D status and RFH-GA, sarcopenia, frailty, or BMI in the bivariate analysis (Table 2).

**Table 2.** Clinical, nutritional, and functional characteristics of patients with advanced chronic liver disease, according to the presence of 25(OH)VitD insufficiency Table 2 long description.

Variable		25(OH)VitD levels		p-Value
	Total	Insufficiency (<30 ng/mL)	Adequate (≥30 ng/mL)	
	*n* (%)	*n* (%)	*n* (%)	
RFH-NPT				0.023
No nutritional risk	15 (31.9)	8 (22.2)	7 (63.6)	
With nutritional risk	32 (68.1)	28 (77.8)	4 (36.4)	
RFH-GA				0.081
No malnutrition	26 (55.3)	17 (47.2)	9 (81.8)	
With malnutrition	21 (44.7)	19 (52.8)	2 (18.2)	
Sarcopenia				0.999
No sarcopenia	23 (54.8)	17 (54.8)	6 (54.5)	
With sarcopenia	19 (45.2)	14 (45.2)	5 (45.2)	
Frailty				0.164
Robust	29 (61.7)	20 (55.6)	9 (81.8)	
Frail	18 (38.3)	16 (44.4)	2 (19.1)	
BMI				0.419
Underweight	5 (10.6)	5 (13.9)	0 (0.0)	
Eutrophic	20 (42.6)	15 (41.7)	5 (45.5)	
Overweight	22 (46.8)	16 (44.4)	6 (54.5)	

BMI: Body Mass Index; MELD-Na: Model for End-Stage Liver Disease-sodium; RFH-NPT: Royal Free Hospital Nutritional Prioritising Tool; RFH-GA: Royal Free Hospital Global Assessment.

Of the 47 participants enrolled, 42 underwent DXA and were included in the sarcopenia analysis. The remaining five patients were excluded from this assessment due to equipment limitations (body weight exceeding device capacity or presence of metallic implants), personal refusal, or clinical deterioration requiring hospitalisation after the initial interview.

After multivariate regression analysis with adjustments (Table 3), patients with higher disease severity according to the Child–Pugh (OR = 8.247; 95% CI: 1.565–43.471; *p* = 0.013) and ascites (OR = 15.382; 95% CI: 1.661–142.488; *p* = 0.016) had increased risk of 25(OH)VitD insufficiency. Among all nutritional and functional parameters evaluated, only nutritional risk identified by the RFH-NPT score remained associated with 25(OH)VitD levels (OR = 13.168; 95% CI: 1.646–105.369; *p* = 0.015).

**Table 3.** Multivariable regression analysis of factors associated with 25(OH)VitD insufficiency in patients with advanced chronic liver disease Table 3 long description.

Variable	OR [95% CI]	*p*-Value
**Liver disease severity**		
Child-Pugh (B/C)^1^	8.247 [1.565; 43.471]	0.013
MELD-Na (≥15)	3.518 [0.781; 15.847]	0.101
MELD 3.0 (≥11)	1.259 [0.253; 6.270]	0.779
Ascites^1^	15.382 [1.661; 142.488]	0.016
**Nutritional status, sarcopenia and frailty**		
BMI (kg/m^2^)^1^	0.857 [0.730; 1.005]	0.058
RFH-NPT (at nutritional risk)^1^	13.168 [1.646; 105.369]	0.015
RFH-GA (with malnutrition)^1^	5.198 [0.854; 31.616]	0.074
With sarcopenia^2^	0.895 [0.217; 3.699]	0.879
Frail^1^	3.344 [0.564; 19.816]	0.184

^1^Multivariable analysis model 1 adjusted for sex, age and aetiology (alcoholic or non-alcoholic).

^2^Multivariable analysis model 2 adjusted for age, and aetiology (alcoholic or non-alcoholic). BMI: Body Mass Index; RFH-NPT: Royal Free Hospital Nutritional Prioritising Tool; RFH-GA: Royal Free Hospital Global Assessment.

ACLD: Advanced Chronic Liver Disease; BMI: Body Mass Index; Child-Pugh: Child-Pugh score; MELD 3.0: Model for End-Stage Liver Disease 3.0; MELD-Na: Model for End-Stage Liver Disease sodium; RFH-GA: Royal Free Hospital-Global Assessment; RFH-NPT: Royal Free Hospital-Nutritional Prioritising Tool; 25(OH)VitD: calcidiol (major circulating form of vitamin D).

## Discussion

The findings of this study confirm the high prevalence of insufficient 25(OH)VitD levels in patients with ACLD. We found that individuals with clinical decompensation (Child-Pugh or ascites) had a significantly higher risk of 25(OH)VitD insufficiency compared to those with less severe disease. Among the nutritional and functional parameters evaluated, only nutritional risk identified by the RFH-NPT score remained significantly associated with vitamin D insufficiency. Taken together, these findings suggest that vitamin D could serve as a complementary biochemical marker in the assessment of ACLD, reflecting both clinical severity and nutritional risk – findings consistent with other authors who observed that serum 25(OH)VitD concentrations significantly decrease as hepatic decompensation (Child-Pugh, MELD scores and hepatic encephalopathy) increases.^(18,19,20,21,22)^ The association between reduced 25(OH)VitD levels and the presence of ascites observed in our study is further supported by the literature.^(23,24)^

This association likely stems from the immunomodulatory role of vitamin D; its deficiency may exacerbate systemic inflammation and contribute to the progression of fibrosis and decompensating events such as ascites and spontaneous bacterial peritonitis.^(24,25,26)^

Thus, there is a clear and strong association between low serum 25(OH)VitD levels and the severity of liver disease. Additionally, 25(OH)VitD insufficiency may accelerate liver disease progression since the activation of VDR exerts an antifibrotic effect.^(22)^

Another notable finding from our study was the association observed between 25(OH)VitD levels and nutritional risk (RFH-NPT) in individuals with ACLD. It is important to contextualise this choice in light of widely used nutritional assessment tools such as the Subjective Global Assessment (SGA), considered one of the most comprehensive subjective methods, and the Global Leadership Initiative on Malnutrition (GLIM) criteria, which represent the current global consensus for malnutrition diagnosis. While both instruments are broadly applicable across clinical populations, our study opted for the RFH-NPT, a disease-specific screening tool validated for individuals with cirrhosis. The RFH-NPT identifies ascites, a hallmark of advanced liver disease that significantly impacts food intake and nutritional status, as a primary criterion for nutritional risk. This ensures greater diagnostic sensitivity in the ACLD population compared to generic instruments, as also suggested by the literature.^(27)^

The absence of a statistically significant association between 25(OH)VitD insufficiency and nutritional status as assessed by the RFH-GA warrants careful interpretation. The RFH-GA is a diagnostic tool that identifies established malnutrition based on objective anthropometric measures (BMI, AC, and TSF) reflecting chronic and already-consolidated nutritional compromise. In contrast, the RFH-NPT is a screening tool that flags patients at risk of nutritional deterioration, with ascites serving as a primary trigger criterion, functioning, in essence, as a ‘red flag’ for imminent nutritional vulnerability in the context of clinical decompensation. This structural distinction likely underlies the divergent associations observed: vitamin D insufficiency appears more closely linked to the early nutritional vulnerability captured by the RFH-NPT than to the overt malnutrition measured by the RFH-GA.

Notably, to our knowledge, no prior study has used the RFH-GA specifically to investigate the association between nutritional status and 25(OH)VitD levels in patients with cirrhosis. By the other side, Tkachenko et al.,^(28)^ in a cohort of 95 patients with liver cirrhosis, reported that malnutrition, assessed by Onodera’s Prognostic Nutritional Index (PNI) – which incorporates serum albumin and total lymphocyte count – was found in 34.7% of decompensated patients and was significantly associated with severe vitamin D deficiency, with a strong correlation between 25(OH)D_3_ and PNI (*r* = 0.679, *p* < 0.001). The reliance of PNI on albumin, a marker that is simultaneously influenced by hepatic synthetic function and systemic inflammation, may amplify its correlation with vitamin D levels in this population, given that both are sensitive to the degree of hepatic decompensation. These findings collectively suggest that the relationship between vitamin D and nutritional status in ACLD is most detectable at the level of nutritional risk rather than at the level of established malnutrition, where multiple overlapping mechanisms may obscure the independent contribution of vitamin D depletion.

Regarding sarcopenia, no significant associations were observed with 25(OH)VitD levels. This contrasts with the findings of Saeki et al.^(9)^ and Okubo et al.^(10)^ who reported higher sarcopenia prevalence in patients with severe vitamin D deficiency. However, in our study, ascites appears to be the primary link between clinical severity (Child-Pugh) and nutritional risk (RFH-NPT) in their association with 25(OH)VitD. Unlike sarcopenia or frailty measures, the RFH-NPT specifically incorporates the presence of ascites as a primary determinant of nutritional risk. This likely explains why the RFH-NPT remained a robust independent predictor of vitamin D insufficiency, whereas other functional markers did not. The persistence of these independent associations reinforces the consistency of our findings, highlighting that 25(OH)VitD is a sensitive marker for the decompensated state and its immediate nutritional consequences, rather than chronic muscle depletion alone.

Based on these results, we suggest that serum 25(OH)VitD has the potential to be used as a marker for disease severity and nutritional status, and its supplementation may reduce the incidence of complications.

### Limitations

This study has a few limitations that should be considered when interpreting the results. First, the sample size (*n* = 47) was not pre-calculated to specifically assess serum 25(OH)VitD levels, which may limit the statistical power to detect associations and restricts the generalisability of the findings. Additionally, the sample consists exclusively of individuals treated within the public healthcare system (SUS) and recruited from a single centre, which may have contributed to a certain homogeneity in the sociodemographic characteristics of the studied population.

Furthermore, while Computed Tomography (CT) is the gold standard for muscle mass assessment, its routine use is often unfeasible in the SUS due to costs and availability. In contrast, DXA is a cost-effective alternative with lower radiation exposure. To minimise measurement bias caused by fluid retention, a critical concern in ACLD, we utilised ASMM. By excluding the trunk, where ascites and oedema accumulate, we provided a more accurate muscle estimate than total lean mass measures. Finally, although we adjusted for age, sex, and aetiology, individual variations in sun exposure (despite the tropical latitude of Maceió, 9°40’S) and hospitalisation periods may influence vitamin D levels and should be considered in future longitudinal studies.

Additionally, DXA was performed in only a subset of participants (*n* = 42 of 47), which may limit the statistical power of sarcopenia-related analyses and the generalisability of these findings within the sample.

### New hypotheses and future directions

The finding of a significant association between low 25(OH)VitD levels and severe liver disease raises important questions about the effectiveness of conventional vitamin D supplementation (D2 and D3) in this population. It is well-known that these forms of the vitamin require hepatic hydroxylation to be converted to the circulating 25(OH)VitD form. Given the compromised liver function in patients with ACLD, this conversion process may be impaired, which could limit the bioavailability and overall effectiveness of standard supplementation strategies.

Corroborating this uncertainty, a comprehensive Cochrane systematic review found no robust evidence that vitamin D supplementation – whether in its parental forms (D2 or D3) or active hydroxylated forms (alfacalcidol or calcitriol) – significantly reduces mortality or clinical complications in adults with chronic liver disease. The review highlighted that most current trials suffer from a high risk of bias and lack of statistical power.^(29)^

## Conclusion

In this study, 25(OH)VitD insufficiency was highly prevalent in patients with ACLD. Ascites and higher Child-Pugh scores (B/C) emerged as the primary clinical predictors of vitamin D depletion, showing 15.4- and 8.2-times higher odds of insufficiency, respectively. Notably, although bivariate analysis showed associations with MELD scores, these did not remain independent predictors in the multivariate model, suggesting that the association is more strongly driven by clinical decompensation and fluid retention.

Furthermore, a robust independent association was identified between 25(OH)VitD insufficiency and nutritional risk (RFH-NPT), with patients showing 13.2 times higher odds of insufficiency. These findings underscore the potential of 25(OH)VitD as a sensitive marker for both clinical decompensation and nutritional vulnerability. While the current results are compelling, further studies are needed to explore the effectiveness of supplementation, particularly with the active form, 1,25(OH)_2_VitD, as a therapeutic strategy to mitigate disease progression.

## Data Availability

This is an unpublished work not under submission process in any other scientific journal. All data is privately accessible.

## Table 1. Long description

A table with 12 rows and 5 columns compares sociodemographic characteristics of patients with advanced chronic liver disease based on 25(OH)VitD levels. The columns are labeled Variable, Total, Insufficiency (<30 ng/mL), Adequate (≥30 ng/mL), and p-Value. The table includes variables such as self-declared race, education, smoking status, comorbidities, alcoholic etiology, diagnosis time, Child-Pugh score, MELD-Na score, MELD 3.0 score, and ascites. Notable trends include a higher percentage of non-white individuals and those with less than elementary school education in the insufficiency group. The presence of ascites and higher Child-Pugh scores are significantly associated with vitamin D insufficiency.

Navigate back to Table 1..

## Table 2. Long description

The table presents data on the clinical, nutritional, and functional characteristics of patients with advanced chronic liver disease, categorized by their 25(OH)VitD levels. It includes three main columns: Total, Insufficiency (<30 ng/mL), and Adequate (≥30 ng/mL), with corresponding percentages. The rows are labeled with different variables such as RFH-NPT, RFH-GA, Sarcopenia, Frailty, and BMI. Notable trends include a higher prevalence of 25(OH)VitD insufficiency in patients identified at nutritional risk by the RFH-NPT (77.8% vs. 22.2%), with a p-value of 0.023 indicating statistical significance. No significant associations were found between vitamin D status and RFH-GA, sarcopenia, frailty, or BMI. The table provides a detailed breakdown of the number and percentage of patients in each category.

Navigate back to Table 2..

## Table 3. Long description

The table presents the results of a multivariate regression analysis, highlighting the odds ratios (OR), 95% confidence intervals (CI), and p-values for various factors associated with 25(OH)VitD insufficiency in patients with advanced chronic liver disease. The table includes two main categories: liver disease severity and nutritional status, sarcopenia, and frailty. Under liver disease severity, the variables are Child-Pugh (B/C), MELD-Na (greater than or equal to 15), MELD 3.0 (greater than or equal to 11), and ascites. Under nutritional status, sarcopenia, and frailty, the variables are BMI (kilograms per square meter), RFH-NPT (at nutritional risk), RFH-GA (with malnutrition), with sarcopenia, and frail. Notable findings include higher odds ratios for Child-Pugh (OR 8.247), ascites (OR 15.382), and RFH-NPT (OR 13.168), indicating an increased risk of 25(OH)VitD insufficiency. The p-values indicate statistical significance for Child-Pugh (p 0.013), ascites (p 0.016), and RFH-NPT (p 0.015).

Navigate back to Table 3..
